# Methamphetamine use and rates of incarceration among street-involved youth in a Canadian setting: a cross-sectional analysis

**DOI:** 10.1186/1747-597X-4-17

**Published:** 2009-08-12

**Authors:** M-J Milloy, Thomas Kerr, Jane Buxton, Julio Montaner, Evan Wood

**Affiliations:** 1 BC Centre for Excellence in HIV/AIDS, St. Paul's Hospital, 608-1081 Burrard Street, Vancouver, BC, V6Z 1Y6, Canada; 2 School of Population and Public Health, University of British Columbia, 5804 Fairview Avenue, Vancouver, BC, V6T 1Z3, Canada; 3 Department of Epidemiology, BC Centre for Disease Control, 655 12th Avenue West, Vancouver, BC, V5Z 4R4, Canada; 4 Department of Medicine, University of British Columbia, 2775 Laurel Street, Vancouver, BC, V5Z 1M9, Canada

## Abstract

**Background:**

Given concerns over rising use of methamphetamine, especially among street-involved youth, and the links between exposure to the correctional system and the production of drug-related harm, we sought to assess the relationship between ever using methamphetamine and reporting ever being incarcerated in the At-Risk Youth Survey (ARYS) in Vancouver, Canada.

**Methods:**

The relationship between ever being imprisoned and ever using methamphetamine was estimated using a multivariate logistic regression analysis while also considering potentially confounding secondary demographic, social and behavioural variables.

**Results:**

Of the 478 youth recruited into ARYS between September 2005 and October 2006, 385 (80.5%) reported ever being incarcerated overnight or longer. In the multivariate model, methamphetamine use was independently associated with ever being incarcerated (Adjusted Odds Ratio: 1.79, 95% Confidence Interval [CI]: 1.03 – 3.13).

**Conclusion:**

Incarceration was very common in this cohort and strongly linked with ever using methamphetamine. This finding is of concern and, along with the previously identified risks of drug-related harm associated with incarceration, supports the development of novel public policy, such as community-based drug treatment, to address the use of methamphetamine among street youth.

## Background

The use of methamphetamine in Western settings is of increasing concern [1,2], especially among street-involved youth [3,4], a vulnerable population already burdened by high levels of morbidity and mortality [5,6]. According to the United Nations Office on Drugs and Crime, methamphetamine now constitutes the second most commonly used illicit drug internationally, second only to marijuana [7].

For older drug users, especially those who use injection drugs (IDU), the dynamics linking drug use, marginalisation and imprisonment are well described [8-10]. Arrest and imprisonment is a common experience, with a history of incarceration reported by at least 75% of participants in community-recruited samples of IDU in Europe [11], Thailand [12] and the United States [13]. Incarceration may be a risk factor for drug related harm among IDU, since exposure to correctional environments has consistently been associated with an increased likelihood of HIV risk behavior and HIV infection [14,15] as well as increased risk of fatal overdose upon release [16].

Sparked by the growing use of methamphetamine and concerns over links to initiation of injection drug use [17], we have previously reported that over 75% of participants in a local cohort of street-involved youth said they had previously used methamphetamine [4]; 25% of all injection initiation experiences involved methamphetamine [4]; and 13% of local overdose events among homeless youth involve the use of methamphetamine [18]. Vancouver is the site of an explosive outbreak of HIV among IDU with current prevalence estimated at 20% [19]; approximately 3% of local street youth are estimated to be HIV-seropositive [20]. Since exposure to the criminal justice system through arrest and incarceration may actually increase drug-related harms [15], we conducted the present study to determine the prevalence of incarceration in a cohort of community-recruited street youth and investigate its relationship with the use of methamphetamine.

## Methods

The At-Risk Youth Study (ARYS) is a prospective cohort of street-involved youth in Vancouver, Canada, that has been described in detail previously [17]. In brief, snowball sampling and street-based outreach were used in an effort to derive a representative sample of street-involved drug using youth. Individuals were eligible for inclusion if they were aged 14 to 26 years old at the baseline interview and had used illegal drugs other than cannabinoids in the previous 30 days. At baseline and every six-month follow-up, participants answer an interviewer-administered questionnaire, are examined by a nurse and provide blood samples for serologic testing. The ARYS study has been reviewed and approved by the University of British Columbia/Providence Research Ethics Board.

For the present analyses, the outcome of interest was reporting ever being incarcerated, or answering "yes" to the question: "Have you ever been in detention, prison, the drunk tank or jail overnight or longer?" The primary explanatory variable of interest was reporting ever using methamphetamine. First, we compared individuals reporting incarceration with those reporting never incarcerated using individual-, social- and structural-level factors we hypothesised could be associated with both the outcome of interest and primary explanatory variable. These secondary explanatory variables included: gender; age; ethnicity (Non-aboriginal vs. aboriginal); education level (< high school vs. ≥ high school); history of foster care (yes vs. no); history of ER use (yes vs. no); hepatitis C virus (HCV) seropositivity (yes vs. no); ever involved in the sex-trade (yes vs. no); ever diagnosed with a mental illness (yes vs. no); ever dealing drugs (yes vs. no); ever being sexually or physically abused (yes vs. no); ever using crack cocaine (yes vs. no); ever using powder cocaine (yes vs. no); ever injecting heroin (yes vs. no); ever using cannibinoids (i.e., marijuana, hashish) (yes vs. no). All drug use variables referred to any prior use.

For univariate analyses, we used Pearson's χ^2 ^test (dichotomous variables) and the Mann-Whitney test (continuous variables) to compare individuals reporting the outcome versus others by the primary and secondary explanatory variables. To fit the multivariate model, we employed a backwards selection procedure we have used previously [21,22]. After beginning with a full model with all covariates included, we fit reduced models, each with one unique secondary explanatory variable removed, and observed in each model the relative change in the coefficient for the term for methamphetamine in the regression equation. We identified the reduced model with the smallest absolute relative change in the methamphetamine coefficient and removed its missing secondary variable from further consideration. The objective of this step is to remove variables with relatively less effect on the value of the coefficient for methamphetamine and, with each step, to preserve variables in the analysis with greater infuence on the value of the methamphetamine coefficient in multivariate analysis. We continued this iterative process until the smallest relative change in the methamphetamine coefficient exceeded 5% of the value of the coefficient. We then fit a final model including methamphetamine use and all remaining secondary explanatory variables as terms in the regression equation.

All statistical analyses were performed in R version 2.6.1 (R Foundation for Statistical Computing, Vienna, Austria). All *p*-values are two-sided.

## Results

Between September 2005 and October 2006, 478 individuals were recruited into the ARYS cohort, of whom 132 (27.6%) were female, 120 (25.1%) reported Aboriginal ancestry and 329 (68.8%) were Caucasian. At the baseline interview, the median age was 22.0 (Interquartile Range [IQR]: 20.0 – 23.9).

Of the 478 participants, 385 (80.5%) reported ever being incarcerated. As shown in Table 1, social and demographic characteristics associated with incarceration in univariate analyses were: older age (Odds Ratio [OR]: 1.23, 95% Confidence Interval [95% CI]: 1.17 – 1.28, *p *< 0.001); having less than a high school education (OR: 1.66, 95% CI: 1.04 – 2.66, χ^2 ^= 4.07 [df = 1], *p *= 0.032); and ever being a victim of abuse (OR: 2.10, 95% CI: 1.32 – 3.34, χ^2 ^= 9.24 [df = 1], *p *= 0.002). Female gender was inversely associated with having a history of incarceration (OR: 0.19, 95% CI: 0.12 – 0.31, χ^2 ^= 48.03 [df = 1], *p *< 0.001). Behavioural and drug-using variables associated with a history of incarceration are shown in Table 2 and included: methamphetamine use (OR: 2.45, 95% CI: 1.53 – 3.90, χ^2 ^= 13.53 [df = 1], *p *< 0.001); crack use (OR: 3.08, 95% CI: 1.89 – 5.03, χ^2 ^= 20.12 [df = 1], *p *< 0.001); cocaine use (OR: 2.49, 95% CI: 1.33 – 4.66, χ^2 ^= 8.02 [df = 1], *p *= 0.003); and drug dealing (OR: 3.19, 95% CI: 1.97 – 5.19, χ^2 ^= 22.03 [df = 1], *p *< 0.001).

**Table 1:** Univariate analyses of social and demographic characteristics associated with reporting ever being incarcerated in ARYS (n = 478)

	**Ever incarcerated**				
Characteristic	No (%)	Yes (%)	Odds Ratio	95% Confidence Interval	*p*-value^1^
**Age (df = 476)**					
Median (IQR)	20.8 (17.7 – 23.4)	22.4 (18.4 – 26.3)	**1.23**	**1.17 – 1.28**	**< 0.001**
**Gender **(df = 1)					
Male	40 (43.0)	306 (79.5)			
Female	53 (57.0)	79 (20.5)	**0.19**	**0.12 – 0.31**	**< 0.001**
**Ethnicity **(df = 1)					
Non-Aboriginal	76 (81.7)	282 (73.2)			
Aboriginal	17 (18.2)	103 (26.8)	1.63	0.92 – 2.89	0.091
**Education **(df = 1)					
≥ High school	38 (40.9)	113 (29.4)			
< High school	55 (59.1)	272 (70.6)	**1.66**	**1.04 – 2.66**	**0.032**
**Foster care^2 ^**(df = 1)					
No	52 (55.9)	183 (47.5)			
Yes	41 (44.1)	202 (52.5)	1.40	0.89 – 2.21	0.147
**HCV status **(df = 1)					
Negative	77 (82.8)	339 (88.1)			
Positive	16 (17.2)	46 (11.9)	0.65	0.35 – 1.21	0.176
**Mental illness^2 ^**(df = 1)					
No	59 (63.4)	225 (58.4)			
Yes	34 (36.6)	160 (41.6)	1.23	0.77 – 1.97	0.378
**Victim of abuse^2 ^**(df = 1)					
No	58 (62.4)	170 (44.2)			
Yes	35 (37.6)	215 (55.8)	**2.10**	**1.32 – 3.34**	**0.002**
**ER use^2 ^**(df = 1)					
No	61 (65.6)	225 (58.4)			
Yes	32 (34.4)	160 (41.6)	1.36	0.84 – 2.18	0.239

1. *p-*values based on χ-square tests of difference (for categorical variables) and the Mann-Whitney test (for continuous)

2. Refers to any instance in the past

**Table 2:** Univariate analyses of behavioural and drug-using characteristics associated with reporting ever being incarcerated in ARYS (n = 478)

	Ever incarcerated				
Characteristic	No (%)	Yes (%)	Odds Ratio	95% Confidence Interval	*p*-value^1^
**Methamphetamine use^2 ^**(df = 1)					
No	42 (45.2)	97 (25.2)			
Yes	51 (54.8)	288 (74.8)	**2.45**	**1.53 – 3.90**	**< 0.001**
**Crack use^2 ^**(df = 1)					
No	37 (39.8)	68 (17.7)			
Yes	56 (60.2)	317 (82.3)	**3.08**	**1.89 – 5.03**	**< 0.001**
**Heroin injection^2 ^**(df = 1)					
No	74 (79.7)	269 (69.9)			
Yes	19 (20.4)	116 (30.1)	1.68	0.97 – 2.90	0.062
**Cocaine use^2 ^**(df = 1)					
No	80 (86.0)	274 (71.1)			
Yes	13 (14.0)	111 (28.9)	**2.49**	**1.33 – 4.66**	**0.003**
**Cannabinoid use^2 ^**(df = 1)					
No	8 (8.6)	19 (4.9)			
Yes	85 (91.4)	366 (93.1)	1.81	0.76 – 4.28	0.169
**Drug dealing^2 ^**(df = 1)					
No	39 (41.9)	71 (18.4)			
Yes	54 (58.1)	314 (81.6)	**3.19**	**1.97 – 5.19**	**< 0.001**
**Sex trade^2 ^**(df = 1)					
No	77 (82.8)	305 (79.2)			
Yes	16 (17.2)	80 (20.8)	1.26	0.70 – 2.28	0.440

1. *p*-value based on results of χ-square test of difference

2. Refers to any time in the past

Results from the final multivariate logistic regression model are displayed in Table 3. The primary explanatory variable, previous use of methamphetamine, was independently associated with ever being incarcerated in a model which included foster care, female gender, Aboriginal ethnicity and crack use. Correlation between the explanatory variables was moderate, ranging from 0.00 to 0.35.

**Table 3:** Multivariate logistic regression analysis of primary and secondary factors associated with reporting ever being incarcerated in ARYS (n = 478)

Characteristic	Adjusted Odds Ratio	95% Confidence Interval	*p*-value^2^
**Methamphetamine use^1 ^**(yes vs. no)	**1.79**	**1.03 – 3.13**	**0.041**
**Foster care^1 ^**(yes vs. no)	1.58	0.94 – 2.65	0.081
**Gender **(Female vs. male)	0.17	0.10 – 0.28	< 0.001
**Ethnicity **(Aboriginal vs. non-Aboriginal)	1.69	0.89 – 3.18	0.107
**Crack use^1 ^**(yes vs. no)	2.45	1.38 – 4.32	0.002

1. Refers to any time in the past

## Discussion

In this survey of street-involved youth in Vancouver, Canada, we observed a high level of both ever being incarcerated and ever using methamphetamine. The level of incarceration observed in this sample (80.5%) is substantially higher than other estimates in surveys of street-involved youth. In 2004, a multi-site cross-sectional study of 1733 Canadian street youth reported 784 (45.2%) had been in jail [23]. A similar level was reported by 536 homeless youth in Portland, Oregon [24]. In our setting, this level of incarceration is higher (80.5% vs. 59.4%) than that observed in a cohort of adult IDU recruited from a local harm reduction facility [15]. Reasons for this heightened level might include, proximally, the prevalence of high-intensity drug use and involvement in the sex trade; and, ultimately, social and structural factors including a dearth of affordable housing and ordnances targeting homeless individuals [25,26].

Although several street youth surveys include contact with the criminal justice system as an explanatory covariate [23,27,28], we are unaware of any study that identifies the factors associated with incarceration among street-involved youth. In the present study, we found methamphetamine use to be independently associated with ever being incarcerated after adjustment for a number of possible social, demographic and behavioural confounders. Since it is not possible to resolve the temporal relationship between the dependent and primary explanatory variable in a cross-sectional analysis, we hypothesise the association is most likely the result of methamphetamine use, and the means required to support it (e.g., sex trade involvement and other criminal activity) increasing the visibility of street youth to police, elevating the risk of arrest and imprisonment. However, the possibility that methamphetamine use is a sequelae of imprisonment for some individuals cannot be excluded. Numerous studies report a shift to higher-intensity drug use, for example the initiation of drug use by injection, upon incarceration [12,29,30]. Similarly, in a sample of 569 street-involved young men who have sex with men in New York City, contact with the criminal justice system was most often found to precede beginning to use drugs such as heroin, cocaine and speed as well as involvement in the sex trade [30]. In a detailed qualitative analysis, Vancouver street-involved youth described the multiple ways methamphetamine use helped them cope with their social and environmental circumstances, including mediating social contacts, maintaining vigilance over themselves and their possessions, and avoiding the use of psychiatric medications [29].

Regardless of whether methamphetamine use is a predictor or sequelae of incarceration, the strong independent association observed between its use and imprisonment in this analysis is cause for concern. As a result of the persistence of drug use by many prisoners [31] alongside the lack of harm reduction and addiction treatment opportunities within correctional environments [31], exposure to correctional environments has been linked to a higher risk for infection with blood-borne pathogens, including HIV, in this setting [15] as others [32,33]. Thus, the frequent imprisonment of street youth who inject methamphetamine could help sustain viral transmission in this population. Although future work should investigate the relationship between contact with police, courts and jails and intake into alcohol and drug treatment programmes for young drug users, the brief sentences typically served by those designated young offenders suggests little rehabilitative care is available [34]. These factors support the development of novel public policies to address methamphetamine use. We recognise that a substantial segment of policymakers as well as the general public supports punitive sanctions for illicit drug use as a signal of social disapproval as well as a disincentive for current or future use. However, we note that little empiric evidence exists of the effectiveness of this approach on either the individual or population level despite the investment of significant public funds [35]. Thus, our findings add support to calls for new policy approaches to curb illicit drug use among members of the population, for example community diversion or expanded access to drug treatment. Some new programmes to address methamphetamine use, especially in the United States, have been developed, including education and public awareness and precursor regulation [36,37]. These initiatives should be rigourously evaluated before being applied to a vulnerable population.

We also observed a high prevalence of ever using crack cocaine in this cohort, with 78.0% of participants reporting ever using the drug. In the univariate analysis, crack cocaine use was strongly associated with ever being incarcerated (*p *< 0.001). While the effect measures of secondary adjusting variables included in confounding models should be interpreted with caution, it is clear that there is a strong and likely independent effect of crack cocaine use increasing the likelihood of incarceration. The link between high-intensity cocaine use and a greater likelihood of drug-related harms, including incarceration, has been well described in this and other settings. Recently, we reported a high level of crack use in this cohort strongly linked with homelessness [38]. Previous research from Vancouver determined that stimulant use, including cocaine and methamphetamine, helps individuals cope with the immediate rigours of street-involved life, including diminishing feelings of hunger, improving wakefullness and awareness and reducing boredom [29,38].

This analysis has some limitations which should be addressed. As random sampling methods could not be employed due to a lack of voters' lists or other registries, findings from this population of street-involved youth might not be generalisable to the entire local street youth population or other settings. However, it is noteworthy the demographic composition of ARYS is similar to other street-youth samples in Vancouver [3,39]. Second, several measures rely on self-report; thus, social desirability bias might have led to an underestimate of the prevalence of some variables. However, we do not believe any bias was differentially reported by history of incarceration. Finally, we were unable to consider the effect of different durations or locations of incarceration nor did we gather information on the age at first incarceration; also, the cohort contains individuals possibly exposed to either youth detention centres, adult facilities, or both. Future research should consider the effect of these modifiers on drug use patterns and other concerns.

## Conclusion

To conclude, this is the first study to describe such high rates of incarceration among street involved youth and to explore risk factors for incarceration among this population. In multivariate regression analysis including several possible confounders, reporting a history of incarceration was strongly associated with ever using methamphetamine. Given the rising prevalence of methamphetamine use in this area as others, and the elevated risk for drug-related harms including HIV infection associated with exposure to correctional environments, these findings support the development of new public policy to support the health of drug-using and street-involved youth, and the exploration of community diversion programs (e.g. addiction treatment) to avoid the high rates of incarceration among this population.

## Competing interests

M-JM, JB, EW and TK declare they have no competing interests. JM has received educational grants from, served as an *ad hoc *adviser to or spoken at various events sponsored by Abbott Laboratories, Agouron Pharmaceuticals Inc., Boehringer Ingelheim Pharmaceuticals Inc., Borean Pharma AS, Bristol-Myers Squibb, DuPont Pharma, Gilead Sciences, GlaxoSmithKline, Hoffmann-La Roche, Immune Response Corporation, Incyte, Janssen-Ortho Inc., Kucera Pharmaceutical Company, Merck Frosst Laboratories, Pfizer Canada Inc., Sanofi Pasteur, Shire Biochem Inc., Tibotec Pharmaceuticals Ltd. and Trimeris Inc.

## Authors' contributions

M-JM and EW conceived the study. EW, TK and M-JM designed the analysis; M-JM performed the statistical procedures. M-JM wrote the manuscript and incorporated all suggestions. JB provided information and edited a draft of the manuscript. JM contributed to the conception and design of the analysis, interpretation of the data and drafting of the report. All authors approved the version to be published.
